# Work environments and HIV prevention: a qualitative review and meta-synthesis of sex worker narratives

**DOI:** 10.1186/s12889-015-2491-x

**Published:** 2015-12-16

**Authors:** Shira M. Goldenberg, Putu Duff, Andrea Krusi

**Affiliations:** Gender and Sexual Health Initiative, British Columbia Centre for Excellence in HIV/AIDS, St. Paul’s Hospital, 608-1081 Burrard Street, Vancouver, BC V6Z 1Y6 Canada; Faculty of Health Sciences, Simon Fraser University, 8888 University Drive, Burnaby, BC V5A 1S6 Canada; Department of Medicine, University of British Columbia, Vancouver, BC Canada

**Keywords:** Sex work, HIV, Sexual risk, Structural determinants, Work environment, Meta-synthesis, Criminalization, Occupational health, Peer support, Third parties

## Abstract

**Background:**

Sex workers (SWs) experience a disproportionately high burden of HIV, with evidence indicating that complex and dynamic factors within work environments play a critical role in mitigating or producing HIV risks in sex work. In light of sweeping policy efforts to further criminalize sex work globally, coupled with emerging calls for structural responses situated in labour and human-rights frameworks, this meta-synthesis of the qualitative and ethnographic literature sought to examine SWs’ narratives to elucidate the ways in which physical, social and policy features of diverse work environments influence SWs’ agency to engage in HIV prevention.

**Methods:**

We conducted a meta-synthesis of qualitative and ethnographic studies published from 2008 to 2014 to elucidate SWs’ narratives and lived experiences of the complex and nuanced ways in which physical, social, and policy features of indoor and outdoor work environments shape HIV prevention in the sex industry.

**Results:**

Twenty-four qualitative and/or ethnographic studies were included in this meta-synthesis. SWs’ narratives revealed the nuanced ways that physical, social, and policy features of work environments shaped HIV risk and interacted with macrostructural constraints (e.g., criminalization, stigma) and community determinants (e.g., sex worker empowerment initiatives) to shape SWs’ agency in negotiating condom use. SWs’ narratives revealed the ways in which the existence of occupational health and safety standards in indoor establishments, as well as protective practices of third parties (e.g., condom promotion) and other SWs/peers were critical ways of enhancing safety and sexual risk negotiation within indoor work environments. Additionally, working in settings where negative interactions with law enforcement were minimized (e.g., working in decriminalized contexts or environments in which peers/managers successfully deterred unjust policing practices) was critical for supporting SWs’ agency to negotiate HIV prevention.

**Conclusions:**

Policy reforms to remove punitive approaches to sex work, ensure supportive workplace standards and policies, and foster SWs’ ability to work collectively are recommended to foster the realization of SWs’ health and human rights across diverse settings. Future qualitative and mixed-methods research is recommended to ensure that HIV policies and programmes are grounded in SWs’ voices and realities, particularly in more under-represented regions such as Eastern Europe and Sub-Saharan Africa.

**Electronic supplementary material:**

The online version of this article (doi:10.1186/s12889-015-2491-x) contains supplementary material, which is available to authorized users.

## Background

Globally, sex workers (SWs) experience a disproportionately high HIV burden across epidemic settings (i.e., in both concentrated and generalized epidemics). A global review of female SWs in 50 countries recently estimated that SWs globally face 13.5-fold higher odds of HIV infection, compared to the general population of women of reproductive age [1]. Studies of male and trans* SWs are scarce, although existing data suggest they also face a greatly elevated HIV burden compared to the general population [2, 3].

Previous work has illustrated that HIV vulnerability and prevention in sex work represent the product of intersecting factors operating at multiple levels of influence. Our team recently published a comprehensive framework for understanding *structural determinants of HIV in sex work,* in which the role of social, policy, and physical features of work environments (e.g., policing, peer interactions, managerial policies and practices, type of work venues); community organization (e.g., community empowerment, sex work collectivisation); and macrostructural determinants (e.g., criminalisation) in shaping HIV epidemics is explicitly highlighted and acknowledged [4, 5]. Such structural determinants interact dynamically with interpersonal (e.g., condom use, types of sexual exchanges, sexual networks), individual behavioural (eg, drug use or duration in sex work), and biological (eg, sexually transmitted disease co-infection) factors to shape vulnerability to HIV acquisition, preventive practices, and transmission risk among SWs [4–7]. In addition to drawing on this structural determinants framework, this review was also underpinned by the concept of *structural vulnerability,* which has previously been useful in framing HIV vulnerability and violence experienced by SWs [8–11]. This includes consideration of how social and structural forces embedded in the organization of society, such as for example, laws, policing, welfare and immigration policies, urban zoning and stigma, render particular groups of people such as SWs, and in particular those living in poverty, disproportionately vulnerable to harm and gives focus to how various structural forces intersect to shape experiences of violence and poor health among SWs [12].

Epidemiological literature highlights the significant variability in HIV risks faced by SWs across different work environments (e.g., indoor versus outdoor venues), yet is often limited in its capacity to elucidate the complex, intersecting and potentially context-specific impacts of work environment features on HIV prevention. Physical features of the work environment (e.g., working in indoor venues, such as brothels, lodges, entertainment venues, hotels, or private homes, versus outdoor spaces such as the street) have often been examined in relation to HIV risk, however this relationship has been found to vary significantly by context. For example, our recent review of the epidemiological literature found that street-based sex work was linked to higher HIV prevalence [13–17] and inconsistent condom use [18–22] in most contexts, while operating in indoor environments such as entertainment venues (e.g., bars, nightclubs, karaoke) was associated with higher HIV risks in some studies [17, 23, 24], but was protective in others [25–31].

Heterogeneity in the relationship between work venue and HIV risks in sex work is likely due to the diversity of working conditions characterizing such environments (e.g., the intersecting influences of policy or social features within different workspaces) – dynamics which are not well captured by epidemiological research designs alone [4, 5, 32]. For example, over 16 studies identified by our recent review of the epidemiological literature indicate that the relationship between venue typology and HIV risk is complex, potentially context-dependent, and greatly heterogeneous, with a need for qualitative research examining the nuanced and intersecting influences of policy, social, and physical features of the work environment [5]. As an example of how qualitative research may be useful in elucidating these intersections, a mixed-methods study among SWs’ clients found that sexual transactions in entertainment venues (i.e., bars, nightclubs) were more likely to be characterized by binge drinking and offers of increased pay for unprotected sex – a finding in seeming contradiction to previous research identifying sex work within indoor venues as more conducive to HIV prevention than outdoor spaces. In mixed-methods analysis, participant narratives characterized indoor entertainment venues as higher-risk than the street due to social norms promoting alcohol use in bars, perceptions of managerial exploitation of bar-based workers, and negative consequences of the enforcement of public health regulations surrounding sex work in bars [33]. These complexities highlight the importance of qualitative research in “unpacking” and elucidating the complex ways in which intersecting work environment features can shape HIV prevention – information that remains essential for informing appropriate occupational health interventions.

Despite a growing body of research showcasing the overall influence of work venue in shaping SWs’ risk of HIV and violence, less is known about the specific features of these environments and the dynamic negotiation of sexual risk and safety across different work environments. Additionally, it remains critical to ensure that policies and programmes aimed at curbing the HIV epidemic are grounded in the voices and lived experiences of SWs across diverse settings and work environments. To gain a better understanding of these lived experiences and to tease out the intersecting influences of physical, social and policy features of work environments, we conducted a synthesis of the qualitative literature on sex work environments and HIV risk and prevention. Given sweeping efforts to further criminalize sex work globally, coupled with emerging calls for structural responses situated in a labour and human-rights framework [5, 7, 34], we reviewed and synthesized qualitative studies published between 2008 and 2014 to describe the nuanced and often intersecting ways in which physical, social, and policy features of work environments (e.g., the ways in which venue policies or managerial practices influence condom use across different types of indoor workspaces) shape SWs’ lived experiences and capacity to mitigate HIV risks.

## Methods

We reviewed and synthesized peer-reviewed qualitative literature published from 2008 to 2014 pertaining to the influence of sex work venues on HIV vulnerability and risk mitigation among SWs (e.g., condom use). Our review and synthesis was informed by guidelines for meta-synthesis of qualitative literature [35] as well as PRISMA guidelines for systematic reviews [36]. We systematically searched the following databases: Pubmed, EMBASE, Science Citation Index, BIOSIS Previews, PsycINFO, CINAHL (Cumulative Index to Nursing and Allied Health Literature), Social Sciences Citation Index, Sociological abstracts, and CAB Direct (CAB Abstracts & Global Health) for studies assessing determinants of HIV or condom use among female sex workers. We used search terms pertaining to sex work (e.g., sex work*, prostitut*), drivers of risk/protective factors (e.g., risk*, context*, vulnerability), and HIV/condom use (e.g., HIV*, AIDS*, condom*, unprotected sex) to retrieve peer-reviewed papers published in any language from January 2008-December 2014. The search was supplemented by cross-referencing and hand-searching key databases (e.g., google scholar).

We initially screened the abstracts and titles for eligibility. All studies among cis- and transgender women SWs were considered for inclusion. Studies focused solely on adolescent SWs (<18 years old), transgender or male SWs, or transactional sex (exchange of sex for non-monetary goods) were excluded. Next, two reviewers (SG and PD) screened the abstracts and full-text papers to assess eligibility for inclusion. Qualitative studies published between 2008 and 2014 that examined physical, social, or policy work environment features in relation to HIV prevention (e.g., condom use) among SWs were eligible for inclusion. We excluded studies that were solely epidemiological (e.g., quantitative analyses) and non-primary research (e.g., reviews, modeling studies, commentaries). Studies in which physical, social, or policy features within the work environment were not a primary focus (e.g., studies focused on community empowerment, macrostructural issues, or individual risks) or which did not present supporting data (e.g., quotes or field note excerpts) were also excluded.

Three independent reviewers extracted data from the different studies, using a standardized form to record details regarding study information (year, setting, study dates), and design (sample size, data collection methods, aim) (Additional file 1: Table S1). Key findings (relationships between work environment features and HIV prevention) were also collected (see Table 1 for summary of key findings ). We also noted major limitations where relevant (e.g., where validity could be impacted by the study design or missing information). The first author oversaw the extraction process, collating and synthesizing the data extracted by each reviewer, and reviewing the included studies and data extractions to ensure consistency across the extraction process. Cases where a lack of clarity or potentially conflicting/inconsistent findings were identified were discussed between the reviewers and the original source was reviewed to verify the information.

**Table 1:** Emergent themes on work environment influences on HIV prevention from sex worker narratives, 2008–2014 (*n* = 24 studies)

Theme	Features that support HIV prevention	Features that undermine HIV prevention
*Occupational health & safety standards in indoor venues*	• Where occupational health & safety are provided in formal indoor spaces, SWs experience enhanced power to negotiate condom use, less violence, access to care, sharing of information and advice [37, 39, 40, 49, 53, 57]	• Operating in isolated, informal spaces often linked to greater susceptibility to violence, barriers to sexual risk negotiation [39, 10] • Enhanced vulnerability in isolated, informal settings can impede sexual health and enhance vulnerability to violence [38, 40, 10, 45–47]
*Influence of third parties*	• Manager support/policies for HIV prevention promotes condom norms, access to information [38, 49] • Protection against client violence by third parties in some indoor venues (e.g., security guards, manager policies on violence) can assist in preventing violence and enables sexual negotiation [37, 41, 42] • Close relationships between managers and workers in managed indoor spaces facilitate condom promotion and access to HIV/STI and health information [56]. • Support from managers/supportive establishment policies available in some informal indoor venues, but often depended on type of relationship [50]	• Lack of manager supports, particularly within criminalized environments results in limited support and a lack of access to condoms, HIV/STI prevention [46, 51, 52] • Manager pressure to service clients quickly/satisfy clients’ needs leads to pressure for unprotected sex. [39, 53] • Concern of manager exploitation or restriction of autonomy relates to reduced control over HIV prevention, extortion for sex [46, 53] • In more informal settings, health protection often left to the individual worker, with a lack of managerial support [50]
*Sex worker/peer support*	• Peer support within the workplace linked to positive outcomes including role modeling, sharing of HIV/STI information, condom negotiation strategies, and support for dealing with difficult clients [49, 56, 57] • Workplaces that promote cooperation, rather than competition, between workers enhanced workers’ power to negotiate condom use and strengthened condom use norms at a venue level [37, 39] • Peers support for HIV prevention also included facilitating access to HIV/STI testing and condoms (e.g., by purchasing in bulk to have available in the venue, lending condoms) [56]	• Lack of social support at work or ability to work with peers related to social isolation, less exposure to advice or information on condom negotiation and sexual health, violence, and enhanced susceptibility to exploitation at work promoted workers’ vulnerability [41, 46, 58]
*Interactions with police*	• Access to police protections and the ability to work without criminalization fostered the creation of trusting relationships with police, ability to report violence, and ability to negotiate sexual transactions without fear of negative consequences [39]	• Harassment, raids, arrest, or detention by police linked to displacement and undermines sexual negotiation [57, 59] • Fear of police harassment, abuse, and arrest and confiscation of harm reduction equipment posed critical barriers to accessing/carrying condoms and other HIV prevention supplies [10, 44] • Direct impacts of police harassment, abuse, and arrest included HIV/STI risks as a result of sexual violence, rape, and sexual abuse by police [45, 46]

We adopted a meta-synthesis approach to systematically integrate and synthesize the results of the 24 articles. Drawing on theoretical principles of structural vulnerability and a structural determinants of HIV in sex work conceptual framework, we used an inductive process to extract key themes of salience to our aim that emerged in each study. Representative quotes which articulated the ways in which physical, social, or policy features of work environments shape SWs’ agency in mitigating HIV risk were explicitly sought and collected for each theme (Table 2). During the synthesis, we considered inconsistencies and potential discrepancies within each theme (e.g., potential for management policies and practices to promote prevention as well as risk), as well as across contexts (e.g., criminalized versus decriminalized policy settings) and regions (e.g., high-income countries (HIC) vs. low- and middle- income countries (LMIC)). As the synthesis progressed, the themes were refined and regrouped, until they yielded a core set of themes which represented key topics identified across studies and settings [35].

**Table 2:** Sex worker narratives: Work environment influences on HIV prevention and risk, 2008–2014 (*N* = 24 studies)

Theme	Features that support HIV prevention	Features that undermine HIV prevention
*Occupational health & safety standards in indoor venues*	*The street just doesn’t appeal to me, because of the whole security safety issues. You know, I like being in a parlour because it is safe. Yeah, sure, you don’t make as much, they take a big cut, but that’s the price you pay, you know, for your health and life. (Indoor parlour)* [37] *The street’s way too dangerous. It’s just so easy for people to do anything they want. Like we’ve lost about, lost two lovely ladies from the street, and you know, just like that, you could just, yeah, just there’s nothing you can do about it when you’re standing out on a corner or any part of the street, there’s nothing much you can do about it. There may be a lot of traffic, but not many people pull over to help. (Street/Independent)* [37]	*[on the street] they [spotters] can take the license plate down and the car make, but once buddy gets you two blocks away, how are they going to stop the guy from shooting or stabbing you? They might prevent it from happening to the next girl, cause they got his plate number, but for you, there’s no protection. None at all. (Street)* [10 ] *He took me to the graveyard. . . There were 5–6 people totally drunk. They did like that only and did not even give me a rupee. They did without condom. (Street)* [39] *They took me by car to a guesthouse and they force me to have sex with four other men. [Do you know if they used condoms?] No. Not used condom because it was a force. (Street)* [47]
*Influence of third parties*	*We meet the boss every Monday and each FEW attends the meeting. Our boss told us in the meeting that the ‘classes’ (intervention sessions) are worth attending. He is really supportive of us taking the classes. After the classes, he often reminds us that health should be our priority and we can make more money if we are healthy. He even jokes that the intervention saves us money to buy condoms and reminds us that no one else outside might be this considerate of us. (Entertainment establishment)* [49] *I explain to the girls that they should use condoms. I emphasize the fact that these people from SHIP are giving us knowledge so that our lives are safe, and we can prevent ourselves from acquiring any such infection or disease. (Madam and SW)* [38] *It is important for my girls to enjoy good health. If she is sick then I need to ensure that she gets medical attention either in our own clinic or outside… I charge her room rent and that is to my benefit; she is getting a room and health care—that is to her benefit. (Madam)* [38] *[I] t is safer with a boss such as when there is a problem, they deal with it. When policeman catch us, they pay for us … They protect us. When people fight us, they also protect us. (Brothel)* [42] *He [the lodge manager] says ‘use condom and do’ but if he [the client] says it’s not fun, then the manager says ‘Take your money and go, we get 100 other clients. (Indoor)* [39] *They [building staff] really pay attention: we get all the lists of all the offenders and stuff, and they’re put up [by the door] and staff study them. One of the staff caught one [a violent client]. He was a visitor in the house, and he came in as a date, and they called the police, and he got arrested. (Indoor – supportive housing)* [40] *Last time, a client wanted oral sex without a condom. I could not persuade him to put one on. Then, I called Manager W from the guest room. The client argued with the manager and said he can get the service in another brothel. Manager W insisted that wearing a condom is regulation here and asked the client to leave politely.(Spa)* [50] *Last time, I put on a condom for a client. But he took it off under the table. Angry, I refused to continue sex with him. I put on my clothes and went downstairs. I know the boss will protect and support me, so I dared to refuse him. (Roadside hotel)* [50] *I am always concerned with their health. I tell them to pay more attention to their personal hygiene. They should always wash their hands after massage, and I tell them to prepare condoms.(Massage Parlour)* [55]	*I got raped and the guy just walked out the door. I was crying and the manager came to me and all he said was, “As soon as you walk in the room, you are on your own. It’s not my problem or my responsibility for your safety. (Indoor parlour)* [52] *Usually, if a man is taking too long my madam starts to blame me and says that I have sat twice, so if he takes a long time I take the condom off. With this customer I convinced him and he discharged quickly so there was no trouble. (Brothel)* [53] *If we sent any client back she used to get angry, so we did not use [condoms] regularly”. (Brothel)* [39] * One day I was harassed by a client and when I told the bar manager, he demanded sex so that he can help me. (Bar)* [46] *Girls from the dalal bari (houses where pimps bring clients) rarely misbehave…The girls there are bound to do all kinds of sex…They rarely can protest or behave badly. For us it is different. If I bring a customer I can make him use a condom or drive him away — I can do that — they cannot do that. (Indoor/Independent)* [53] *The boss never talks about condom use. He pretends he doesn’t know [the sex business]. There is no talk about condom use or HIV prevention among girls either. Nobody talks about this. We only talk about which client is rich or which is decent (Massage Parlour) *[55]. *It is better to train the girls. The boss doesn’t care about us. He has his business to take care of. He never talks about this with us (Sauna)* [55]
*Sex worker/Peer support*	*When I first came here to start working, it was [another SW] who taught me how to use condoms. She said that this was the hygienic way *[57]. *In the waiting room, new FEWs will stay with old FEWs. We don’t feel as embarrassed to discuss these things (sex and condoms) in the venue compared to when we are outside. (Entertainment venue)* [49] *Clients prefer to spend hours talking and drinking before taking a girl for sex…we observe and consider the clients and check references with friends. (Entertainment venue) *[41]. *Sometimes mammies would ask us to show pity for new girls who were in the same room with us and teach them things they didn’t understand. In this case, we would tell them a lot of things and teach them a lot. (Indoor)* [49] *When my friends [sex workers] knew when or where there were free services for HIV or STI testing and counseling, they called me and asked me to go there with them. (Indoor)* [56] *My friend [sex worker] encouraged me to use a condom when I had my first client. She told me to use condoms to protect myself from being infected. [Furthermore,] I was too shy to buy condoms at first. My friend [sex worker] bought them for me and taught me how to use them. (Indoor)* [56] *My FSW friends advised me that using condoms could prevent diseases (Indoor) *[56]. *When I get into a conflict with clients, my colleagues will come to mediate the conflict and persuade the client to use a condom (Indoor).* [56]	*I don’t talk to friends about my genital symptoms because friends who are jealous of me might disclose it to clients. Also I may be asked to stop working for a while, or even not be allowed to work in this bar if the bar owner knows about this.I wait and quietly visit a health clinic alone. (Entertainment venue)* [41] *I felt tidak pantas (inappropriate). Even though I do what they [other sex workers] do, I will never associate myself with them. I will not share anything with them; I just feel it isn’t appropriate. From day one I felt that someone here disliked my presence. (Brothel)* [58] *During my first weeks there, no one told me to use condoms or even offered me a condom. I didn’t know what condoms looked like, so when I had clients I just did it, simple and crazy, really crazy. (Brothel)* [58]
*Interactions with police*	*On the corner, doing it in the car, I used to be scared all the time, paranoid about cops, scared about getting charged… It’s a lot easier now. I can come and go, and cops actually say hi to me. (Indoor – supportive housing)* [40] *It’s safer. I can just yell for help, and, you know, in the alley you can’t really yell, you know? It’s hard to run away, and… you don’t know whether they’re going to get violent or something. There’s a lot more chance of that outside than at my place. [If it happened in my room] I’d run for the door. It’s happened before, and the staff have come, and they told him to leave, or they even got the police to get him to leave. They do that right away. It took 4 cops to get this guy to leave. Then they barred him [from the place]. (Indoor – supportive housing)* [40]	*We faced many problems as we stood on the streets; mainly from the police and the local goons. Police used to dump us in the vans, ask for Rs. 60,9 and used to have sex with us. They used totake money as well as service. The local goons used to charge…They used to say, “You people stand here and earn well, so you have to give something to us.” If we said no, they would beat us up. See here, they once stabbed my hand with a knife [showing a large scar on her hand]. (Street)* [38] *They mistreat us so much. Even policemen rape us, and if you try to resist, they threaten you with arrest and detention *[59]. *[The police] look in your purse and if they find condoms, they put you in jail, but clients are released. (Street)* [44] *The difficulties were great, especially when we started. The local police dispatched people to drive us out. They didn’t understand. They would take our condoms and throw them on the groun *[57]. *They’re not out to get us, but they don’t really have any compassion or concern about us. A lot of us girls start carrying pepper spray or bear spray. But you have to be careful too, because as soon as the cops search you, jack you up, they take away what you can to protect yourself with, even rigs. (Street)* [10]

## Results

Of 1514 articles screened for eligibility, we identified 24 eligible qualitative studies that elucidated the nuanced and often intersecting ways in which features of work environments (e.g., establishment managerial practices, access to security, peer roles, policing) shape SWs’ capacity to engage in HIV prevention. Of the 24 studies, 19 were from LMICs, primarily Asia (*n* = 14) (e.g., China, India) and Sub-Saharan Africa (*n* = 5); five studies were from HICs (e.g., Canada, New Zealand). Characteristics of the studies included are described in Additional file 1: Table S1.

Although most of the studies emphasized work environment features that undermined HIV prevention (e.g., working in isolated, outdoor spaces; negative interactions with police; workplace violence), SWs’ narratives also highlighted key work environment features that enhanced their agency to successfully negotiate condom use (Tables 1 and 2). SWs’ narratives revealed the critical influence of occupational health and safety standards in indoor establishments, as well as the extent to which third parties engaged in protective practices (e.g., support for condom use; intervening in violent encounters) within such venues. Occupational health and safety was often perceived as most effectively facilitated in workplaces where there was access to SW/peer support (e.g., ability to share advice on condom use, strategies for dealing with difficult clients), as well as in contexts where negative interactions with police were minimized (e.g., working in decriminalized contexts or environments in which peers/managers successfully deterred unjust policing practices).

### “I like being in a parlour because it is safe”: Occupational Health and Safety Standards in Indoor Workspaces

Across settings, indoor workspaces (e.g., in-call venues, managed spaces such as massage parlours) characterized by workplace health and safety standards often featured prominently in participants’ narratives as critical for condom negotiation and access to HIV prevention (e.g., condoms, HIV/STI testing). SWs’ narratives elucidated how condom use with clients could be more safely and effectively negotiated in indoor workspaces where occupational standards, policies and protections were in place [37, 38]. In India, Canada, and New Zealand, formal indoor workspaces typically offered a greater degree of protection (e.g., safety mechanisms; the presence of other workers, managers, madams, or other staff who could deter or assist in the removal of violent or uncooperative clients). Where such protections were offered, workers generally experienced enhanced agency and opportunities for safer sex negotiation [37–40]. As the narrative of a parlour-based worker from New Zealand highlighted:

*The street just doesn’t appeal to me, because of the whole security safety issues. You know, I like being in a parlour because it is safe. Yeah, sure, you don’t make as much, they take a big cut, but that’s the price you pay, you know, for your health and life.*

*(Sex worker, Indoor parlour, New Zealand)* [37]

While working in informal indoor spaces such as entertainment venues (e.g., bars, nightclubs, beer gardens) was often perceived as safer than outdoors, this varied widely due to the lack of occupational standards and substantial diversity of informal venues [39, 40]. In comparison to more formal and managed spaces, operating out of unfamiliar and informal settings was often linked to reduced control over clients, the terms of transactions, substance use, and condom use [39, 40]. An emergent theme in entertainment venues related to alcohol use with clients, which was a common feature of the social environment. While this was perceived by most entertainment-based workers as having some benefits in terms of providing opportunities to observe and screen clients prior to negotiating a transaction (e.g., as an entertainment-based worker in Laos noted, “*Clients prefer to spend hours talking and drinking before taking a girl for sex…we observe and consider the clients and check references with friends”)* [41], working in alcohol-serving venues was also noted to undermine SWs’ agency to negotiate condoms within the context of client intoxication or pressure to use alcohol themselves [42, 43]. As an entertainment-venue based SW in Cambodia explained, “*When the alcohol gets in, he always requests me not to use condom”* [42].

SWs’ narratives suggested that outdoor settings were often the least conducive to HIV prevention, with street-based workers reporting being pushed to the margins due to fear of policing and violence [42, 10]. Operating in dark, isolated areas typically undermined SWs’ access to security while working [10], leading to an increased risk of physical and sexual violence [38, 40, 10–47] and limiting opportunities for effective condom negotiation with clients [38, 39]. As the narratives of SWs from New Zealand and Canada highlighted the dangers of outdoors and how this limited opportunities to ensure safety and use condoms consistently:

*The street’s way too dangerous. It’s just so easy for people to do anything they want. Like we’ve lost about, lost two lovely ladies from the street, and you know, just like that, you could just, yeah, just there’s nothing you can do about it when you’re standing out on a corner or any part of the street, there’s nothing much you can do about it. There may be a lot of traffic, but not many people pull over to help.*

*(Sex worker, Street/Independent, New Zealand)* [37]

*Out there [street/public location] you’re like a hostage almost. You feel almost that bad if you were out there. You’re going to settle, or you’re going to put yourself in a bad position maybe. If it’s not going good, you’re stuck, and that’s not a good feeling. You don’t want to be isolated…*

*(Sex worker, Unsanctioned indoor workspace, Canada)* [40]

For those working outdoors, a lack of access to familiar and safe spaces to service clients was a key concern. For example, SWs’ narratives suggested that servicing clients in unfamiliar or isolated locations (e.g., clients’ homes, hotels, vehicles) represented situations which elevated the risk of violence (e.g., gang rape, forced unprotected sex, lack of payment) due to the lack of agency this provided SWs in controlling the context and terms of a transaction: [40, 45, 46]

*Once you get into a guy’s house, they can just…That’s it, you know. You don’t know where you’re gonna go. At a guy’s place, it’s more or less like, what he says you have to do, you know. Um. I guess you just go with uh, risk.*

*(Unsanctioned indoor workspace, Canada)* [40]

*Sometimes when they went in a different direction, I opened the door and jumped out of the car…when he takes you in a different direction from the hotel, there is a chance of gang rape.*

*(Sex worker, Street, Thailand)* [48]

### “I know the boss will protect and support me”: Third Parties as Protective of SWs rights

The extent to which indoor workspaces facilitated HIV prevention was greatly shaped by the extent to which third parties engaged in protective (vs. exploitative or more 'hands-off') practices. In Cambodia, workers’ narratives suggested that some, but not all brothel managers implemented policies supportive of condom use [42], whereas SWs in entertainment venues rarely received condoms from establishment managers [42]. In India, Canada, Cambodia and China, SWs highlighted the importance of working in establishments where HIV prevention and education (e.g., provision of condoms at work, managers discussing and establishing norms for condom use) were promoted, insofar as managers were respectful of their agency and human rights, as opposed to taking a punitive or coercive approach [38, 39, 42, 49].

For example, in Belgaum, India, brothel-based SWs operate as more permanent staff, with transactions controlled by a brothel madam. In this context, the policies and practices put in place by the madam strongly determined SWs’ earnings, types of clients, and services performed. SWs indicated that there was great variation in such practices across madams and brothels, with some reporting exposure to exploitative practices that undermined HIV prevention, whereas in cases where SWs’ autonomy was respected and positive occupational health policies were adopted, this often enhanced SWs’ agency in HIV prevention [39]. In light of this, a key pillar of the community-led Sonagachi HIV/AIDS Intervention Program (SHIP) and subsequent community-led initiatives of the large-scale Avahan Project in India involved incentivizing brothel madams to support HIV prevention (i.e., by ensuring that brothels were safe and viable workspaces) [38]. As brothel-based SWs and madams participating in the SHIP project described the positive effects of such an approach:

*No, I will never have sex without a condom. Not worth it. I kick them out, and if they don’t go, I call for help.*

*(Sex worker, Brothel, India)* [38]

*I explain to the girls that they should use condoms. I emphasize the fact that these people from SHIP are giving us knowledge so that our lives are safe, and we can prevent ourselves from acquiring any such infection or disease.*

*(Manager, Brothel, India)* [38]

SWs’ narratives revealed that when third parties engaged in protective practices, including practices to promote safety (e.g., bad date sheets, security guards, cameras, managers intervening in situations of client violence), this could successfully reduce their vulnerability to violent encounters while fostering opportunities to negotiate sexual risk reduction with clients [37, 40–43, 50]. As SWs’ testimonials from Cambodia, China and Canada highlighted:

*There’s cameras on each floor, they’re not allowed in unless they have ID, their name is written down, and, people have seen you with the guy, so he knows that he can’t go and try to do something to me and get away with it.*

*(Sex worker, Unsanctioned indoor workspace, Canada) *[40]

*[I]t is safer with a boss, such as when there is a problem, they deal with it…They protect us. When people fight us, they also protect us.*

*(Sex worker, Brothel, Cambodia)* [42]

*Last time, I put on a condom for a client. But he took it off under the table. Angry, I refused to continue sex with him. I put on my clothes and went downstairs. I know the boss will protect and support me, so I dared to refuse him.*

*(Sex worker, Roadside hotel, China)* [50]

Although the protections afforded by third parties in indoor workspaces were described as critical for supporting SWs’ health and safety, their implementation was often limited by macrostructural constraints, including sex work-related stigma and criminalization (e.g., of third parties) in most of the contexts studied. For example, stigma and concerns regarding frequent raids and fear of arrest within criminalized settings often discouraged managers from engaging in protective practices, such as discussing condom use in the workplace or keeping condoms onsite [51], and in some settings constrained management from interfering in situations of violence [52]. In these cases, managers often preferred a ‘hands-off’ management style to avoid implicating their establishment in illegal activities – which was perceived by SWs’ to constrain agency to refuse client pressures for unprotected sex or to protect oneself from or report client-perpetrated violence [51, 52]. As a Canadian SW explained:

*I got raped and the guy just walked out the door. I was crying and the manager came to me and all he said was, “As soon as you walk in the room, you are on your own. It’s not my problem or my responsibility for your safety.”*

*(Sex worker, Massage parlour, Canada)* [52]

Additionally, SWs in Asia and Sub-Saharan Africa frequently reported establishment practices and policies that prioritized profit over their workers’ health and safety or that constrained workers’ autonomy, resulting in negative impacts on SWs’ health and working conditions [39, 46, 50, 53, 54]. For example, in a number of studies from India and China, participant narratives included experiencing pressure to accept client requests for unprotected sex due to managerial practices that prioritized client satisfaction and financial considerations over occupational health: [39, 53–55]

*Usually, if a man is taking too long my madam starts to blame me and says that I have sat twice, so if he takes a long time I take the condom off.*

*(Sex worker, Brothel, India)* [53]

*The boss does not care about condom use. She only cares about earning money. She is very kind to sex workers who do not use condoms because they bring her more clients.*

*(Sex worker, Indoor venue, China) *[56]

Although norms supportive of condom use often existed among managers in indoor settings, their role in shaping condom negotiation between workers and clients was complex and varied substantially across venues and contexts. For example, an ethnographic study involving SWs and managers in China highlighted the role of managers as both intermediaries and protectors of SWs, who can facilitate or impede agency depending on the type of relationship they maintain with their workers [55]. Similarly, a qualitative study in three Chinese cities elucidated how although maintaining healthy workers was often perceived to be important for the success of managers’ businesses, to retain and attract clients, managers’ goal was to ensure their customers were satisfied, which could lead to contradictory practices with respect to HIV prevention [56]. Some studies indicated that in more upscale indoor venues and venues where managers and workers maintain closer and more trusting relationships, managers may be more likely to develop policies and practices to protect sexual health, even at the expense of interfering with their business [50]; whereas in venues where employee turnover was high (e.g., due to high mobility of SWs and unfavorable working conditions), managers were often less invested in workers’ wellbeing and were less engaged in ensuring workplace health and safety [55]. This dichotomy is illustrated below:

*I am always concerned with their health. I tell them to pay more attention to their personal hygiene. They should always wash their hands after massage, and I tell them to prepare condoms.*

*(Manager, Massage Parlour, China)* [55]

*The boss never talks about condom use. He pretends he doesn’t know [the sex business]. There is no talk about condom use or HIV prevention among girls either. Nobody talks about this. We only talk about which client is rich or which is decent.*

*(Sex Worker, Massage Parlour, China)* [55]

SWs’ narratives also indicated that managers could contribute to abusive practices that enhanced vulnerability to HIV (e.g., sexual abuse, exploitative working conditions) [46, 53]. For example, as a bar-based SW in Kenya shared her experience: “*One day I was harassed by a client and when I told the bar manager, he demanded sex so that he can help me” *[46]. Concerns of exploitation by managers, a desire for increased flexibility and autonomy, and a preference to keep more of one’s earnings (rather than having to pay a room/management fee) meant that in some contexts, SWs described a preference to work independently. The following narratives highlight the nuances of independent versus managed indoor sex work in terms of their relative advantages and disadvantages:

*I don’t like having a boss. I don’t want to be under their control. They benefit from my sweat. If by myself, I can decide to do or not to do … If we are independent, we can do anything we want. No-one controls us.*

*(Sex worker, Street, Cambodia)* [41]

*Yes, I have (thought of working in other sectors), but I didn’t want to. I found the street more freely to work, but just it was just dangerous at the same time, but I was more free when I worked out on the, as a street worker than what I would be inside, cause there’ll be rules and regulations, yeah, and I’m not really used to rules and regulations and people telling me what to do.*

*(Sex worker, Street, New Zealand)* [37]

### “We stand up for each other”: Access to Sex Worker/ Peer Support

SWs’ narratives across the diverse settings studied articulated the ways in which SW/peer support within the workplace was shaped by management practices and occupational policies, as well as the broader legal context of sex work. Occupational health and safety was often perceived as most effectively facilitated in indoor spaces where trust and mutual support amongst workers could be cultivated [41, 56]. Within such workspaces, SWs often shared advice on condom negotiation and use, as well as strategies for preventing or dealing with difficult/violent clients, fostering a supportive atmosphere for HIV prevention in some contexts [40, 41]. As entertainment and brothel-based workers in China highlighted the importance of SW/peer support, particularly for migrants and newcomers to the sex industry: [49, 57]

*We talked about condoms, boyfriends and things like that, such as ‘condoms can protect us’ and ‘we may be infected by HIV if we don’t use condoms.’ Sometimes there would be arguments among us. Some girls said they never used them and others said that we must use them.*

*(Sex worker, Entertainment venue, China)* [49]

*My friend [sex worker] encouraged me to use a condom when I had my first client. She told me to use condoms to protect myself from being infected. [Furthermore,] I was too shy to buy condoms at first. My friend [sex worker] bought them for me and taught me how to use them.*

*(Sex worker, Indoor venue, China)* [56]

*When I get into a conflict with clients, my colleagues will come to mediate the conflict and persuade the client to use a condom.*

*(Sex worker, Indoor venue, China)* [56]

In India, SW/peer support was also noted to be important for facilitating SWs’ ability to leverage their collective power to stand up against exploitative or abusive practices such as exploitative managerial practices, pimping, or violence perpetrated by police or clients [38]. The narratives of participants in the Sonagachi project in India highlighted these as positive impacts of the SHIP intervention, elucidating the reciprocal pathways by which peer support can influence other features of the work environment (e.g., management practices in brothels):

*We help each other in the brothel. The atrocities and the harassment inflicted on us within the locality…the thugs who would harass us and would hit the young girls… sometimes the madam would not give some of the girls their due share of the money. If any such beating and harassing happens, then we stand up for each other, we support each other and we put a stop to it.*

(*Sex worker, Brothel, India)* [38]

While many studies identified peer support as critical for countering risks and setting norms for condom use at a venue level, stigma and criminalization often undermined the cultivation of supportive relationship and practices among SWs [46]. Some studies within indoor workspaces reported on management practices that fostered competition (rather than trusting or supportive relationships) among SWs and undermined discussions of condom use and safety among workers [39, 41, 53, 58]. In Indonesia, limited peer support between SWs in indoor spaces was often attributed to the intersecting influences of economic competition (e.g., competition for clients or preferential treatment by management) and other macrostructural factors, such as stigma and migration/mobility patterns, with newcomer/migrant SWs often perceived as a ‘threat’ and thus excluded from social networks. At the same time, newcomer/migrant SWs’ experiences indicated that internalized stigma (i.e., self-stigma associated with perceptions of sex work as ‘immoral’) further deterred them from associating with peers in the workplace, exacerbating social isolation and undermining social solidarity and access to peer support mechanisms [58].

*I felt tidak pantas (inappropriate). Even though I do what they [other sex workers] do, I will never associate myself with them. I will not share anything with them; I just feel it isn’t appropriate. From day one I felt that someone here disliked my presence.*

*(Sex worker, Brothel, Indonesia)* [58]

Moreover, SWs’ narratives in some settings (e.g., Cambodia, Canada) suggested that peer support (e.g., peer health and safety mechanisms) varied widely between indoor venues, rendering workers in some informal spaces (e.g., guest houses, bars) more isolated and vulnerable to violence, and constraining their capacity to negotiate condom use [42]. In Canada, frequent police harassment and fear of arrest among street-based SWs was perceived to undermine their ability to access peer-based safety and support mechanisms (e.g., peers acting as spotters), primarily as a result of displacement away from established work environments [40, 10]. Importantly, peer support was often perceived as less available in contexts where sex work and illicit drug use scenes overlapped to a greater extent (e.g., on the street and in informal spaces):

*It’s more about the drugs and stuff down here [on the streets], like, nobody really helps anybody down here unless you have dope.*

*(Sex worker, Street, Canada)* [40]

### “I used to be scared of getting charged”: Interactions with Police

Across settings and regions, fear of arrest, harassment, or abuse by police increased structural vulnerability to HIV, constituting common barriers to sexual health and safety. In addition to health-promoting establishment policies and peer supports, an important feature of supportive workspaces was reduced vulnerability to negative interactions with law enforcement, and in some cases, improved relations with police. In Canada and India, SWs in supportive indoor venues noted how these models could promote more trusting relationships with police. Participant narratives highlighted how this often facilitated improved health, safety, and access to justice, such as by fostering SWs’ agency in reporting violent incidents, abuse, or threats in the workplace to police [38, 40]. As a Canadian SW discussed the ways in which staff and police within her workplace were able to assist in removing violent clients, which she contrasted against the isolation and lack of agency she associated with street-based work:

*It’s safer. I can just yell for help, and, you know, in the alley you can’t really yell, you know? It’s hard to run away, and… you don’t know whether they’re going to get violent or something. There’s a lot more chance of that outside than at my place…It’s happened before, and the staff have come, and they told him to leave, or they even got the police to get him to leave. They do that right away. It took 4 cops to get this guy to leave. Then they barred him [from the place].*

*(Sex worker, Supportive indoor environment, Canada)* [40]

In India, the Sonagachi project and other community empowerment initiatives (e.g., the Ashodoya intervention in Mysore) emerged as another example of improved relationships SWs were able to achieve with police; this was accomplished through SWs using their collective power to stand up against unjust practices and abuses by police, and to promote more cooperative relations with police at an establishment level (e.g., engaging managers to stand up against police) [38, 39]. SWs’ narratives indicated that one the key outcomes of the Sonagachi project was the mobilization of SWs against police harassment, abuse, and other unjust law enforcement practices:

*We go door to door to these brothels when word comes in that a sex worker has been arrested and we need to gherao the police station, or organize a rally to protest harassment.*

*(Sex worker, Brothel, India)* [38]

*Sometimes when a sex worker is picked up by the police, then SHIP comes to us and we [fellow brothel residents] immediately go to the police station to protest.*

*(Sex worker, Brothel, India)* [38]

Despite the importance of these examples, interventions that have successfully mitigated harassment and other unjust police practices have been limited to only a few settings. In most countries - particularly those lacking strong SW-led community empowerment movements - the potential benefits of such models remain constrained by the persistent criminalization and stigmatization of sex work. In most of the settings studied, SWs’ narratives instead emphasized the significant harms of punitive policing practices such as the confiscation and use of condoms as evidence of illegal activities. In Cambodia, Kenya, Uganda, and Canada, SWs feared carrying or accepting condoms from health workers as a result of policing [42, 10–45, 51, 57, 59]. These practices were constrained SWs’ capacity to engage in HIV prevention in most settings, with the exception of the decriminalized setting of New Zealand [10, 44, 46, 60]. As a SW from Kenya described her experience:

*[Police] look in your purse and if they find condoms, they put you in jail.*

*(Sex worker, Street, Kenya) *[44]

SWs’ narratives across a range of formal and informal work environments, such as entertainment venues in Asia and massage parlours in North America, also elucidated how police crackdowns and surveillance in criminalized environments limited access to condoms [42, 51]. Such actions displaced workers away from settings characterized by strengthened HIV prevention practices, towards more unsafe workspaces where agency to negotiate condoms was much more constrained. In Cambodia, displacement to informal spaces such as guest houses often resulted in enhanced HIV risks for SWs compared to the supports that had fostered condom use in brothels (e.g., access to peers, supportive venue policies) [42]. In Kenya and Canada, street-based SWs’ experiences of displacement to more isolated spaces due to policing often resulted in having to rush the screening of potential clients – a critical step used by SWs to ensure safety and negotiate the use of condoms upfront [10, 44]. In some contexts, human rights abuses by police (e.g., extortion, sexual abuse) were so pervasive that this was described as a normalized aspect of SWs’ daily working life – a perception that was sometimes perceived as legitimized by the criminalization of sex work [46]. This was most commonly documented in Sub-Saharan Africa, where human rights abuses by police, such as rape and forced sex to avoid arrest/detention, directly contributed to structural vulnerability [45, 46, 59].

*They mistreat us so much. Even policemen rape us, and if you try to resist, they threaten you with arrest and detention.*

*(Sex worker, Street, Uganda)* [59]

## Discussion

This synthesis elucidated the nuanced and complex impacts of physical, social, and policy features of work environments on SWs’ agency to negotiate HIV prevention across a range of settings and regions, as well as their intersections with broader macrostructural constraints (e.g., criminalization, stigma) and community determinants (e.g., SW/peer empowerment initiatives). Drawing on a structural determinants conceptual framework as well as theoretical concepts of structural violence, this synthesis identified four main broad themes elucidating the influence of workplace social, physical, and policy features on HIV prevention and vulnerability*.* Across workspaces and settings, participant narratives highlighted how structural vulnerability and agency to engage in HIV prevention is shaped by working conditions such as (1) occupational health and safety standards, (2) access to third party protections, (3) opportunities to work collectively with peers, and (4) initiatives to address/remove punitive legal environments surrounding sex work. The findings support calls for multi-level interventions, including scaling-up models of occupational health and safety where SWs’ human and labour rights are promoted, as a cornerstone of effective HIV prevention [5, 61]. Support for community empowerment and peer outreach, interventions that engage third parties as partners in HIV prevention, and strategies to shift away from punitive legal approaches (e.g., decriminalization, police education programmes to align law enforcement and HIV prevention priorities) represent promising strategies warranting future investigation [5, 61, 62].

This review highlights the critical need for additional qualitative research and mixed-methods research among diverse contexts to ensure that local, national, and international HIV policies and programmes in sex work are grounded in SWs’ voices and realities. Additionally, given that many of the studies identified emphasized primarily risky, rather than supportive, features of work environment, mixed-methods studies examining specific features that confer resilience across a more diverse range of settings remains needed. The design and testing of safer workplace interventions is an especially important avenue for future research in Eastern Europe and Sub-Saharan Africa, where we identified a particular dearth of information.

While a number of prominent work environment themes emerged from our analysis, their manifestations varied regionally, often reflecting their interplay with broader macro-structural determinants and community empowerment initiatives [37, 40, 53]. Indoor settings where occupational standards, health-promoting practices of third parties, and peer supports were available often supported SWs’ safety, rights, and agency to negotiate condoms; however, the extent to which indoor venues were health-promoting hinged strongly on local legal and human rights conditions. For instance, within the decriminalized context of New Zealand, managers often encouraged HIV prevention and occupational safety [37], whereas within criminalized settings, such as massage parlours in Canada, managers often faced legal constraints to supporting HIV prevention (e.g., police raids) [52]. Despite evidence that managers can (and often do) play a critical role in HIV prevention, they are in many settings criminalized through legislation to combat trafficking for sexual labour, which inadvertently increased structural vulnerability and undermined SWs’ health and safety. For example, in Canada, third parties have been historically criminalized, and continue to be through new legislation that prohibits the purchase of sex and criminalizes third parties who economically profit from sex work (i.e., the *Protection of Communities and Exploited Persons Act*) [63]. Clear evidence indicates that legislation criminalizing aspects of the exchange of sex between consenting adults severely constrains SWs’ vulnerability to violence and poor sexual health [64]. Supporting opportunities to consensually work with peers and third parties (e.g., security, managers) and legal approaches that avoid conflation of sex work and trafficking remain critical for more effectively supporting SWs’ health and human rights [65].

In this meta-synthesis, participant narratives strongly emphasized the positive implications of peer support for enabling HIV prevention. These findings largely reflect investments in community empowerment initiatives in Asia, which foster supportive managerial practices, peer supports, reductions in stigma, and in some cases, improved relations with police [38, 49, 57]. In regions lacking strong community empowerment initiatives (e.g., Sub-Saharan Africa), SWs’ narratives indicated that criminalization, stigma, and exploitative third party practices limited peer support and agency to negotiate sexual risk [46, 58]. For example, SWs in Kenya, South Africa, Uganda and Zimbabwe, described how violence and human rights abuses (e.g., unlawful arrests and detention, sexual violence, extortion by police) undermined health and safety; although SWs in these settings recognized the benefits of unified action to counter risks in their work environment, criminalized and stigma often limited such collectivization [46]. Community empowerment interventions have been associated with reductions in HIV/STIs and sexual risks in India, the Dominican Republic, and elsewhere [62], often through their effects on access to supportive workspaces (e.g., opportunities for collective action, improved police relations, reduced stigma). The promise of such models for promoting safer work environments and reductions in HIV/STIs across a broader variety of settings (e.g., Sub-Saharan Africa, Eastern Europe) warrants future research and scale-up.

Lastly, this review highlighted the heterogeneity of sex work environments, as well as the complex pathways through which work environment features shape the experiences of SWs negotiating HIV prevention within these spaces. The findings highlight the need for mixed-methods research (particularly to evaluate safer work environment models), given that such complexities may not be well captured by epidemiological methods alone. Comparative research across geographic, epidemic, and policy settings, including settings in which sex work is decriminalized, is needed to identify the most effective approaches and pathways by which different work environment models influence HIV prevention, particularly in heavy-HIV burden settings of Sub-Saharan Africa and Eastern Europe.

This synthesis built on a recent comprehensive review of the epidemiological evidence on structural determinants of HIV in sex work [5], to gain a better understanding of the nuanced and intersecting impacts of work environment features on SW’s agency in negotiating HIV prevention with their clients. To our knowledge, this is the first attempt to systematically review and synthesize this body of work. Not all of the studies reviewed clearly delineated the pathways by which work environment characteristics influenced SWs’ capacity to mitigate HIV risk, despite our best efforts to identify these studies. Additionally, studies were excluded which were not peer-reviewed or published prior to 2008, or which did not have an explicit work environment focus (e.g., studies of economic determinants, individual risk factors, or community empowerment determinants which lacked a work environment focus).

## Conclusion

Findings of this meta-synthesis highlight the imperative to promote ‘enabling environments’ that support SWs' agency to negotiate HIV prevention with their clients within the workplace – including access to formal/in-call indoor workspaces with occupational standards, supportive third party practices that promote HIV prevention and safety, access to peer/sex worker support, and protection from criminalization. While these common themes were identified across settings, their manifestations often varied regionally, reflecting their interplay with broader macro-structural determinants (e.g., the criminalization of sex work) and community empowerment initiatives. To foster the realization of SWs’ health, human and labour rights, policy reforms and community initiatives to remove punitive approaches to sex work, ensure supportive workplace standards and policies, and foster SWs’ ability to work collectively are recommended across diverse settings. Future qualitative and mixed-methods research is recommended to ensure that HIV policies and programmes in sex work are grounded in SWs’ voices and realities, particularly in more under-represented regions such as Eastern Europe and Sub-Saharan Africa.

## Additional file

Additional file 1: Table S1. Description of Qualitative Studies Included in Meta-Synthesis, 2008-2014 (*n* = 24 studies). (DOCX 50 kb)
