# Supplementary material for: Work environments and HIV prevention: a qualitative review and meta-synthesis of sex worker narratives
Source: BMC Public Health. 2015 Dec 16;15:1241. doi: 10.1186/s12889-015-2491-x (PMC4681074; doi:10.1186/s12889-015-2491-x)
Supplement: Additional file 1: Table S1. — Description of Qualitative Studies Included in Meta-Synthesis, 2008-2014 (n = 24 studies). (DOCX 50 kb) [file 12889_2015_2491_MOESM1_ESM.docx]

| **Table 1: Description of Qualitative Studies Included in Meta-Synthesis, 2008-2014 (n=24 studies)** | | | | | |
| --- | --- | --- | --- | --- | --- |
| **Author and Publication Year** | **Setting** | **Study Dates** | **Study Aim** | **Data Collection Methods** | **Sample population of sex workers** |
| Abel and Fitzgerald, 2012[36] | Auckland, Wellington, Christchurch, Nelson, Napier, New Zealand | Unavailable | To explore movement of SW between sectors of sex industry after decriminalization | Survey  In-depth interviews | n=772 (survey)  n=58 (interviews) |
| Bungay et al., 2012[53] | Metro Vancouver, BC, Canada | 2004-2006 | To understand experience of violence among women working in indoor sex venues; similarities and differences in Canadian-born and immigrants | Questionnaires  Individual and group interviews | n=21 |
| Buzdugan et al., 2012[38] | Belgaum, Karnataka, India | 2009 | To understand venue-based vulnerabilities | In-depth Interviews | n=50 |
| Chen, 2012[49] | Shanghai, China | Unavailable | Examining interventions to diffuse HIV prevention information through SWs venues | In-depth interviews | n=21 |
| Evans et al., 2008[54] | Kolkata, India | 1995-1997 | To describe the role of human agency, context and structure in HIV prevention | In-depth interviews  Focus group discussions | n=61 |
| Ghose et al., 2011[37] | Kolkata, India | 2006-2008 | To examined the living and  working conditions of brothels involved in the Sonagachi Project | In-depth interviews | n=50 |
| Handlovsky et al., 2012 [52] | Greater Vancouver Regional District, southwest BC, Canada | 2004-2006 | To assess women’s experience with condom use and how these are situated within the social dynamics of their work in the indoor sex industry | Questionnaires  Individual and group interviews | n=21 |
| Hampanda 2013 [44] | Mombasa, Kenya | 2007 | To contextualize the selling of sex and the challenges of practicing safe sex among female SWs | In-depth interviews  Focus group discussions | n=28 |
| Hao et al, 2015[56] | Qingdao, Heifei, Nanning, China | 2012 | To describe the roles of various components (i.e., relations, structures and functions) of female SWs’ networks and their influence on condom use | In-depth interviews  Focus group discussions | n=116 |
| Hong et al, 2014[51] | B city, Guangxi region, China | 2008 | To describe the relationship between female SWs and their gatekeepers (managers) | In-depth interviews | n= 38  n=16 managers |
| Januraga et al, 2014[58] | Denpasar and Badung District, Bali, Indonesia | 2013 | To elucidate how the interaction between social and environmental factors shape mobility and HIV risk among newcomer SWs | In-depth interviews | n=32 |
| Jie et al, 2012[55] | Guangzhou, China | Unavailable | To explore SW history, condom use, HIV testing service, barriers to condom u se | In-depth interviews | n=24 |
| Krusi et al. 2012 [39] | Vancouver, BC, Canada | 2009-2010 | To assess how unsanctioned indoor sex work environments influence safety and risk negotiation with clients during sex work transactions | In-depth interviews  Focus group discussions | n=39 |
| Liu et al. 2014[50] | Liuzhou, China | 2009 | To understand how organizational culture is reflected in artifacts, beliefs and underlying assumptions of venue | Participant observation  In-depth interviews | n=33 |
| Maher et al, 2013[47] | Phnom Penh, Cambodia | 2007-2010 | To explore young female sex workers’ accounts of negotiating condom use with a range of sexual partners | In-depth interviews | n=33 |
| Maher et al, 2011[41] | Phnom Penh, Cambodia | 2007-2010 | To explore relationships between sex work contexts and HIV/STI vulnerability, and to identify situational and structural impediments to HIV prevention | In-depth interviews | n=33 |
| Mbonye et al. 2013 [42] | Kampala, Uganda | Mar 2010 – June 2011 | To explore locations, types of women involved and interaction with clients for women who provide sex | In-depth interviews | n=58 |
| Okal et al. 2011 [45] | Naivasha & Mombasa, Kenya | 2007 | To examine the social and legal contexts that underpin the high levels of sexual and physical violence pervading sex work in Kenya | Focus group discussions | n=81 |
| Phrasisombath et al., 2012[40] | Savannakhet province, Laos | 2010 | To explore the working environment and perceived risks and benefits among female SWs | In-depth interviews  Focus group discussions | n=39 |
| Ratinthorn et al[48] | Bangkok, Thailand | Unavailable | To explore characteristics of violence against street SWs and how violence influences personal and societal health risks | In-depth interviews  Focus group  Field observations | n=28 |
| Schoemaker and Twikirize, 2012[59] | Kampala, Uganda |  | To explore SWs’ perception of risk of HIV infection in context of serious threats | Peer ethnography  In-depth interviews | n=68 |
| Scorgie et al., 2013[46] | Mombasa, Kenya; Bulawayo, Zimbabwe; Kampala, Uganda; South Africa | Dec 2010 and Jan 2011 | To illustrate the combined effects of criminalization and law enforcement on social relations and how this affects health and wellbeing | In-depth interviews  Focus group discussions | n=106 |
| Shannon et al. 2008[43] | Vancouver, BC | Dec 2005 to Mar 2006 | To explore the role of social and structural violence and power relations in shaping the HIV risk environment and prevention practices of women in survival sex work | Focus groups | n=46 |
| Tucker et al, 2011[57] | South China (various cities) | 2009-2010 | To examine social networks among female SWs and determine potential for sexual health promotion | In-depth interviews | n=34 |
